# Immune and neurotrophin stimulation by electroconvulsive therapy: is some inflammation needed after all?

**DOI:** 10.1038/tp.2015.100

**Published:** 2015-07-28

**Authors:** E M van Buel, K Patas, M Peters, F J Bosker, U L M Eisel, H C Klein

**Affiliations:** 1 Department of Molecular Neurobiology, Center for Life Sciences, University of Groningen, Groningen, The Netherlands; 2 Department of Nuclear Medicine and Molecular Imaging, University Medical Center Groningen, University of Groningen, Groningen, The Netherlands; 3 Institute of Neuroimmunology and Multiple Sclerosis, Center for Molecular Neurobiology, University Medical Center Eppendorf, Hamburg, Germany; 4 Department of Psychiatry, University Medical Center Groningen, University of Groningen, Groningen, The Netherlands

## Abstract

A low-grade inflammatory response is commonly seen in the peripheral blood of major depressive disorder (MDD) patients, especially those with refractory and chronic disease courses. However, electroconvulsive therapy (ECT), the most drastic intervention reserved for these patients, is closely associated with an enhanced haematogenous as well as neuroinflammatory immune response, as evidenced by both human and animal studies. A related line of experimental evidence further shows that inflammatory stimulation reinforces neurotrophin expression and may even mediate dramatic neurogenic and antidepressant-like effects following exposure to chronic stress. The current review therefore attempts a synthesis of our knowledge on the neurotrophic and immunological aspects of ECT and other electrically based treatments in psychiatry. Perhaps contrary to contemporary views, we conclude that targeted potentiation, rather than suppression, of inflammatory responses may be of therapeutic relevance to chronically depressed patients or a subgroup thereof.

## Electroconvulsive therapy

Major depressive disorder (MDD) is one of the major causes of disability in the Western world, accounting for 6% of the total burden of disease in Europe as measured by loss of disability-adjusted life years.^1^

The pathogenesis of MDD is elusive. This is testified by the number of hypotheses articulated over the years, which have taken into account perturbations in monoamine metabolism, neuroendocrine function, glutamatergic neurotransmission, hippocampal neurogenesis and overall neurotrophic support.^2, 3, 4, 5^ Yet, one of the latest additions to the puzzle—the inflammatory theory—aspires to bring these pieces together.^6, 7^

Electroconvulsive therapy (ECT)—the induction of convulsive seizures via epicranial electrodes placed unilaterally or bilaterally—is one of the most effective treatment strategies for MDD, showing superior efficacy compared with antidepressant medication in numerous studies.^8^ One of the main indications for ECT is treatment-resistant depression, in which it can reach remission rates of up to 50%.^9^ In addition, as its onset of action is much faster than for conventional antidepressants, ECT may be a suitable choice in patients with a high suicide risk requiring immediate clinical improvement.^10^ Furthermore, there are indications that off-label use of ECT may be beneficial in other neuropsychiatric disorders as well, including schizophrenia, Parkinson's disease and Huntington's disease.^11, 12, 13^

Despite this range of action, the mechanisms by which ECT exerts its beneficial effects remain largely unknown. Lately, however, animal studies have demonstrated that electroconvulsive seizures (ECS, the animal model for ECT) induce structural changes within the brain at the cellular and molecular levels. Of particular interest is the observation that both ECS and ECT induce several changes in neurotrophin and immune signaling, both in the central nervous system (CNS) and in peripheral tissues. This might explain the effect range of ECT, as all conditions that have been reported to improve by ECT have been linked to immune dysregulation and/or neurotrophic deficits.^11, 12, 13, 14, 15, 16, 17, 18, 19, 20^

The immune and neurotrophic systems influence each other in complicated ways that are just beginning to be understood. This paper attempts a synthesis of our knowledge on the neurotrophic and immunological aspects of ECT.

## Neurotrophic aspects of ECT

### ECT enhances hippocampal neurogenesis

The subgranular zone of the hippocampus is one of the few sites in the adult mammalian brain where neurogenesis takes place. Several lines of preclinical evidence associate MDD with impaired neurogenesis (reviewed in Miller and Hen^21^). Indirect evidence from human studies is in line with the neurogenic theory of MDD. For instance, magnetic resonance imaging studies have shown a decrease in hippocampal volume in MDD patients, which correlated with the duration of illness.^22^ Moreover, there is evidence of hippocampal dysfunction in MDD, resulting in memory impairment.^23^ The neurogenic theory of MDD is further reinforced by the finding that the behavioral effects of antidepressants are largely dependent on their ability to stimulate hippocampal neurogenesis in animal models.^24^ Importantly, there is now direct preclinical evidence for the role of impaired neurogenesis in the emergence of a depressive phenotype.^25^

Preclinical studies further show that chronic administration of ECS is associated with an increased number of hippocampal granule cells^26^ and granule cell mossy fiber sprouting.^27^ The neurogenic effect of ECS is stronger than that of pharmacological antidepressants, and the onset is faster, being comparable to the fast onset of clinical improvement upon ECT in MDD patients.^26^ Direct evidence of ECS-induced neurogenesis comes also from studies in nonhuman primates.^28^ In humans though, a neurogenic effect of ECT can only be indirectly deduced by studies showing a volumetric increase in the hippocampus.^29^ Of note, ECT-induced volumetric changes in humans are not specific to this anatomical region,^30^ suggesting that brain plasticity mechanisms beyond neurogenesis may also be involved in the action of ECT.

### ECT induces BDNF upregulation

Hippocampal neurogenesis is regulated by a variety of neurotrophic factors (reviewed in Lee and Son^31^). One of the most studied neurotrophic factors is brain-derived neurotrophic factor (BDNF). The ‘neurotrophic hypothesis' of depression postulates that MDD may result from stress-induced decreases in BDNF and homologous factors within CNS networks critically involved in the pathophysiology and/or treatment of the disorder.^32^ Indeed, antidepressants increase hippocampal BDNF levels and this increase is thought to be critical for their therapeutic effects.^33, 34^ In addition, BDNF administration into the hippocampus induces neurogenesis and has antidepressant effects in animal models of depression.^35^ These antidepressant-like effects may be mediated by altered sensitivity to stress, as the sensitivity to stress-induced depression-like behavior has been shown to be related to hippocampal BDNF expression in mice with altered expression of the glucocorticoid receptor.^36^

Several lines of evidence demonstrate that ECT alters BDNF levels and/or BDNF signaling, suggesting that this neurotrophin may be involved in the antidepressant effects of ECT as well. In rodents, ECS increases BDNF mRNA and protein in cortical and hippocampal areas.^37, 38, 39^ In addition to BDNF, ECS upregulates mRNA expression of the BDNF receptor, TrkB (tyrosine receptor kinase B), in several cortical and hippocampal areas^39^ as well as intracellular signaling cascades activated by TrkB, such as the Ras–Raf–MEK–ERK pathway and the PI3K/Akt pathway.^40, 41, 42^ These pathways stimulate a variety of intracellular processes, including processes involved in the regulation of proliferation and survival.

Interestingly, numerous clinical studies have demonstrated reduced *peripheral* levels of BDNF in untreated MDD patients compared with both antidepressant-treated patients and healthy controls.^43^ Although it is generally believed that these findings are peripheral manifestations of the neurotrophic hypothesis, experimental data from rodent studies show that even widespread elevations of central BDNF are not necessarily reflected in the periphery.^44^ Furthermore, neurotrophins are widely expressed in non-neuronal tissues,^45^ thereby further complicating the use of blood BDNF as a proxy marker for central processes. Most importantly, animal studies have demonstrated that *peripherally* administered BDNF is rapidly taken up by CNS tissues^46^ and exerts both neurogenic and antidepressant-like effects,^47^ strongly suggesting that central changes of BDNF levels and/or signaling may be, in part, driven by peripheral BDNF fluctuations. In line with this possibility, Sartorius *et al.*^48^ suggested that blood-borne BDNF contributes to parenchymal BDNF after repeated ECS in rats.

In patients, several studies observed increased serum or plasma BDNF levels after ECT,^49, 50, 51, 52, 53, 54^ whereas others have found unaltered or decreased levels.^55, 56, 57^ The difference in outcome may be due to the difference in the time lag between treatment and blood sampling. In general, in studies that found increased BDNF levels upon ECT, this time lag was longer than in studies that did not find such an effect, indicating that although ECT does increase peripheral BDNF levels, these levels may only reach their maximum in the circulation 1 week to 1 month after completion of therapy. This view is in line with a recent study in MDD patients undergoing ECT, showing that the increase in peripheral BDNF levels is positively correlated to both seizure quality markers as well as the interval between the last ECT session and the blood withdrawal.^58^ The authors suggested that this might be due to a delayed (>6 days) and increased equilibrium of peripheral BDNF that is secondary to an early central rise of the neurotrophin. This interpretation, however, is not mutually exclusive with the possibility that peripheral sources of BDNF are concurrently mobilized by ECT.

### ECT induces VEGF and angiogenesis

Another factor believed to be important in ECS-induced neurogenesis is vascular endothelial growth factor (VEGF). VEGF stimulates neuronal proliferation via its receptor, fetal liver kinase 1 (Flk-1).^59, 60^

VEGF infusions directly increase the number of neuronal progenitor cells in the rat hippocampus.^61^ Importantly, ECS-induced neuronal proliferation can be blocked by inhibition of VEGF-Flk1 signaling, indicating that VEGF is indispensable for ECS-induced neurogenesis.^61^ Animal studies have further shown that VEGF has antidepressant-like properties.^62^ However, it is unclear whether these antidepressant-like properties are causally related to the neurogenic effects of VEGF. Alternatively, VEGF-induced antidepressant-like effects may be related to neuronal plasticity. Indeed, it has been demonstrated that memory-related effects of VEGF are mediated by synaptic plasticity rather than neurogenesis.^63^ As reduced synaptic plasticity is believed to be related to symptoms of depression as well,^64^ the ability of VEGF to stimulate neuronal plasticity may have a role in its antidepressant-like effects.

VEGF is also a potent stimulator of angiogenesis. This is of interest as hippocampal angiogenesis is closely linked to neurogenesis.^65, 66^ In fact, it is believed that most neurotrophic factors possess at least some angiogenic properties. Angiogenesis may be essential for the supply of nutrients and other blood-borne growth factors necessary for ECT-induced neurogenesis. It is also possible that proliferating endothelial cells are an additional non-neuronal source of growth factors during ECT.^67, 68^

In patients, serum VEGF was demonstrated to be increased upon ECT and this increase correlated with clinical improvement,^69^ further suggesting that VEGF is an important component of the antidepressive efficacy of ECT.

## Immunological aspects of ECT

### Rapid stimulating effects of ECT on circulating cytokines

Increased cytokine mobilization in the peripheral blood, for example, increased levels of tumor necrosis factor-alpha (TNF-α) and interleukin-6 (IL-6), is a common immunological finding in MDD patients^70, 71^ or a subgroup thereof.^72^ Inflammation-related genes have also been found to be upregulated in postmortem frontal cortex of medication-free MDD patients,^73^ suggesting focal inflammatory processes in the CNS.

Numerous preclinical studies in rodents as well as clinical studies in patients undergoing treatment with interferon-alpha have suggested a role for inflammatory cytokines in MDD.^6, 74^ However, one must keep in mind that the intensity of endogenous inflammation seen in the peripheral blood of MDD patients is comparably much less pronounced than that seen in classical inflammatory, autoimmune or interferon-treated disorders.^75^

Perhaps counterintuitively, single ECT induces a transient (15–30 min) increase in the expression of pro-inflammatory cytokines, such as TNF-α, IL-1β and IL-6.^76, 77^ Acute ECT was also found to render peripheral blood monocytes of MDD patients more sensitive to a proliferating stimulus (lipopolysaccharide), as shown by a more enhanced secretion of TNF-α and IL-6 from these cells.^78^ Importantly, these cytokine elevations were observed in patient monocytes upon both the fifth and the eleventh of a series of ECT sessions, suggesting that this short-term pro-inflammatory component of ECT is integral to every session and is not moderated throughout repetitive treatments.

However, Hestad *et al.*^77^ followed ECT-treated MDD patients using a longer longitudinal protocol and showed that although ECT indeed increases TNF-α 1 h after the first session, repeated treatments gradually reduce TNF-α levels. For proper interpretation, it should be noted that the observed reduction of plasma TNF-α in this study was most pronounced 1 week after the *last* ECT session, thereby precluding acute effects of the electrostimulus on cytokine measures. In addition, the majority of the patients had clinically responded to ECT by that time point.

Thus, overall, individual ECT sessions acutely upregulate circulating inflammatory cytokines, suggesting an immediate and strong induction of systemic innate immune responses, possibly associated with robust somatic manipulations. On the other hand, multi-session ECT may over time result in the normalization of peripheral blood cytokine measures. Nevertheless, it is unclear whether such a normalization results from a direct suppressing effect of the treatment on the immune system or whether it is merely secondary to clinical remission.

### Stimulating effects of ECT on peripheral innate immune cells

In terms of cellular immune parameters, numerous clinical studies have shown in the past that MDD patients may exhibit relatively increased numbers of neutrophil granulocytes (neutrophilia) as well as signs of functional immunosuppression, as exemplified by reduced mitogen-induced T-cell proliferation and reduced natural killer cell cytotoxicity.^79^ It is thus interesting to note the effect of ECT on such parameters.

Fluitman *et al.*^78^ showed that acute ECT (15–30 min after the electrostimulus) induces a leukocytosis in MDD patients, driven by significant increases in absolute numbers of granulocytes, monocytes and natural killer cells. By contrast, T cells were reduced in absolute counts. A similar leukocyte pattern of polymorphonuclear leukocytosis and relative lymphopenia was observed 2 h after a single ECT in a previous study.^80^ When a longer interval was used, mitogen-induced proliferative responses of lymphocytes were also found decreased after repeated ECT.^81^

Although overall reductions in lymphocyte counts and proliferative responses seem to be associated with ECT, the percentage and absolute numbers of *activated* T cells were found increased upon completion of another ECT study in MDD patients.^82^ Furthermore, there are consistent indications that natural killer cell activity is transiently but significantly boosted in MDD patients upon both single and repeated ECT.^78, 82, 83^

Animal studies seem to recapitulate some of the observations of the human studies, especially the stimulating effects on the monocyte and neutrophil compartments. For instance, chronic treatment with ECS has been reliably shown to induce a sustained increase in proliferation and metabolic activity of rat peritoneal macrophages as well as lipopolysaccharide-stimulated mixed splenocytes.^84, 85^ A marked increase of phagocytic activity was also evident in rats following focal repeated electrical stimulation of the hippocampus.^86^ Intriguingly, the innate cellular response to electrical CNS stimulation is not only seen in peripheral tissues but also in the CNS vasculature, as increased trafficking of blood-derived macrophages (but no CNS infiltration) has been observed in hippocampal vessels following repeated ECS in rats.^87^

### Stimulating effects of ECS on microglial activity

Microglia—the resident macrophages of the CNS—take charge in the active immune surveillance of the healthy brain and respond accordingly to changes in the microenvironment. They are, therefore, considered the most sensitive sensors of changes in CNS homeostasis (reviewed in Graeber and Streit^88^). Accordingly, one would expect an enhanced responsiveness of these cells to ECT.

Indeed, studies in rodents have consistently shown that ECS increases glial proliferation in several brain areas, including the hippocampus, amygdala, prefrontal cortex and hypothalamus.^89, 90, 91, 92^ Although most studies suggested that these cells remain in an inactive state, two studies have demonstrated changes indicative of increased microglial activity after ECS.^89, 93^ Jansson *et al.*^89^ have shown increased numbers of activated microglia as early as 2 h following the last of a series of ECS. Microglial activation was transient in most CNS areas; however, in the hippocampus, the number of activated microglial cells remained increased for up to 4 weeks after ECS. These results coincide with the study of Jinno and Kosaka,^93^ who have found reduced microglial process density in the hippocampus 24 h after a single or repeated ECS. One month after ECS, microglial process density was still decreased in the repeated ECS group, but not in the single ECS group. Retraction of microglial processes is commonly associated with microglial activation, and therefore these results likely indicate increased microglial activity.

Thus, while some studies indicate that ECS does not influence microglial activity,^90, 91, 92^ other studies suggest that there is an effect on microglial activity.^89, 93^ A reason for this discrepancy may have been methodological. Although Wennström *et al.*^90, 91, 92^ and Jinno and Kosaka^93^ both based their results on morphological examination; the microglial marker used for this examination differed between these studies. Instead, Jansson and co-workers investigated the presence of markers specific for activated microglia.^89^ In addition, Jinno and Kosaka^93^ used different electrical intensities during the ECS treatments than the one used in the other studies, and considering that they reported several mice dying during ECS, one might question whether the intensity chosen was perhaps too high and might have resulted in CNS damage. Moreover, the species that was used (mice or rats) and the time point at which animals were killed differed between these studies.

A pro-inflammatory effect of electrical fields propagating in the CNS has also been demonstrated in a rat model of transcranial direct current stimulation.^94^ In specific, an increase of proliferating cells and upregulation of activated microglia in the cortex ipsilateral to the stimulation site was evident following daily administration of transcranial direct current stimulation for 5 consecutive days. Importantly, this early innate immune response was not associated with cortical lesions or astrogliotic scarring.

## The immune and neurotrophin systems mutually influence each other

### Immune cells produce neurotrophins in an activation-dependent manner

Intriguingly, BDNF and its corresponding receptor TrkB are widely expressed by lymphoid organs and virtually all major subsets of immunocompetent cells (see for reviews: Tabakman *et al.*^95^ and Vega *et al.*^96^). Most importantly, literature from the field of neuroimmunology points to a generalized increase in the availability of humoral neurotrophins, including BDNF, in response to immune stimulation. For instance, both human and rodent peripheral blood mononuclear cells (that is, lymphocytes, natural killer cells, monocytes) constitutively transcribe BDNF mRNA and secrete neuroactive BDNF protein, while producing significantly enhanced levels of the neurotrophin upon both antigen-specific and nonspecific stimulation.^97, 98, 99^ Furthermore, IL-6 and TNF-α are able to stimulate BDNF secretion from human monocytes in a dose-dependent manner.^100^ Accordingly, a positive association between peripheral IL-6 and BDNF has been recently shown to exist in a subgroup of MDD patients, but not in non-depressed controls.^101^

Interestingly, immune-cell derived BDNF is considered to have a protective role in neuroimmunological disorders such as multiple sclerosis and CNS injury,^95, 99, 102^ and this notion has been extended to psychiatric disorders.^103^ Indeed, clinical data indicate that leukocyte BDNF gene expression is decreased in MDD patients,^104, 105^ whereas serum BDNF restoration and clinical improvement in these patients are paralleled by increases in leukocyte BDNF expression following antidepressant treatment.^106, 107^

It is currently unknown whether ECT/ECS specifically upregulates BDNF expression in peripheral blood leukocytes. However, it is tempting to hypothesize that the generalized immune stimulation induced by this treatment (see sections ‘Rapid stimulating effects of ECT on circulating cytokines' and ‘Stimulating effects of ECT on peripheral innate immune cells') renders the innate immune system a vector of peripheral BDNF increases. Such increases may in turn contribute to central enhancement of BDNF (see section ‘ECT induces BDNF upregulation').

In support of this hypothesis, a recent report shows that the CNS and peripheral leukocytes are equally affected by transcranial magnetic stimulation, a non-convulsive modality of brain stimulation which involves induction of intracranial electrical currents by externally applied magnetic fields. Although much less invasive than ECT, repetitive transcranial magnetic stimulation was able to enhance BDNF-TrkB signaling in the CNS as well as in peripheral lymphocytes.^108^ This effect was confirmed in both animals and human subjects and the magnitudes of activation in the two anatomical sites were significantly correlated. Of note, this study once more suggested that transcranial magnetic stimulation-induced upregulation of plasma BDNF is not driven by central BDNF ‘spillover' to the periphery.

### Neurotrophins stimulate immune function

Despite their name, neurotrophins can also be seen as potent autocrine- or paracrine-acting immunotrophins, with multiple functions in the circulation as well as in lymphoid organs. For instance, BDNF can modulate cytokine expression in human peripheral blood mononuclear cells,^109^ as well as in the bone marrow microenvironment.^110^ In addition, BDNF was shown to increase survival of mouse thymocyte precursors.^111^ Similarly, impaired B cell development was observed in BDNF-deficient mice^112^ and conditional deletion of BDNF in T cells and macrophages resulted in reduced T-cell activation and cytokine production.^102^

The immunostimulant properties of neurotrophins can also be seen in the rodent CNS, as microglia-derived BDNF can have a positive autocrine effect, promoting further microglial activation.^113^ However, neuron-derived BDNF negatively affects the antigen-presenting potential of microglia,^114^ suggesting that the central immune effects of neurotrophins are tightly regulated *in vivo*.

### Stimulated microglia may exhibit neuroprotective and antidepressive properties

It is noteworthy that microglia-derived BDNF has been shown to stimulate axonal regeneration in the context of experimental spinal cord injury^115^ or exert long-term neuroprotection via sustained neurogenesis in an animal model of stroke.^116^ However, prolonged or out-of-proportion exposure to microglial activation may pave the way for inflammation-mediated neurodegeneration (reviewed by Correale^117^).

This ambivalent character of microglial activation seems to be dictated by the degree of needs arising in the microenvironment. For instance, Lai and Todd^118^ demonstrated that primary microglia stimulated neuronal survival after exposure to media from moderately injured neurons. This effect was not observed after exposure to media from mildly or severely injured neurons. Interestingly, classical pro-inflammatory cytokines were upregulated by microglia only in response to mild injury, whereas BDNF was upregulated in response to all degrees of neuronal injury.

The beneficial potential of microglial activation in the context of MDD has been recently demonstrated in a translationally relevant study that used a chronic unpredictable stress paradigm in rodents.^119^ In particular, it was shown that after an initial short period of microglial activation, chronic stress leads to subsequent hippocampal microglial apoptosis, decline in cell numbers and dystrophic morphology. In addition, higher suppression of the microglial compartment following chronic stress was associated with higher suppression of neurogenesis and greater depressive-like behavior.

Strikingly, peripherally induced microglial activation (for example, by an acute intraperitoneal injection of lipopolysaccharide) had a dramatic neurogenic effect in the hippocampus, produced an overall increase in microglial cell numbers and reversed the depressive-like phenotype of chronically stressed animals.^119^ Although neurotrophin assessments were not reported in this study, it is very likely that the neurogenic and antidepressant-like consequences of microglial stimulation were, at least in part, mediated by an activated neuroprotective microglia phenotype.

The perspective of antidepressant-like effects mediated by inflammatory stimulation of the chronically challenged CNS is corroborated by both preclinical and clinical studies showing that broadly used nonsteroidal anti-inflammatory drugs may negatively interfere with the mode of action and the efficacy of clinically used antidepressive strategies, such as first-line antidepressive medications^120, 121^ and deep-brain electrical stimulation.^122^

In contrary to popular belief, the above-mentioned findings suggest that potentiation—rather than suppression—of pro-inflammatory responses may be of therapeutic relevance to chronically depressed patients or a subset thereof. Of note, disease chronicity is associated with higher endogenous inflammation and metabolic dysregulation in antidepressant-treated MDD patients.^123, 124^ Given that ECT is usually a second-line intervention reserved for refractory MDD, it is conceivable that the patients amenable to this treatment are—despite peripheral inflammation—in a prolonged state of microglial suppression elicited by disease chronicity. In light of the study by Kreisel *et al.*,^119^ higher peripheral inflammation in these chronically depressed patients could be reconceptualized as an allostatic attempt of the peripheral innate immune system to stimulate microglia-derived repair and antidepressant capacities in the CNS. This, however, would inevitably come about at the expense of somatic health.^125^

## Concluding remarks

Maintenance of bodily tissues depends on *graded* inflammatory responses—what differentiates advantageous from pathological inflammation is the intensity and the timing of its appearance.^126^ This is particularly relevant to the maintenance of CNS plasticity during both health and disease.^127^ The findings mentioned above are well in line with the neuroprotective properties of inflammation that have been extensively described in the general field of neuroimmunology.^128^ We believe this calls for a balanced appreciation of the significance and the role of inflammation in psychiatric disorders as well.

**Figure 1:**
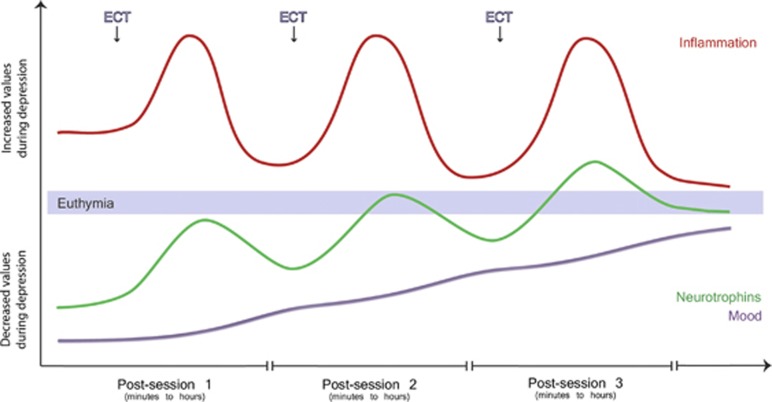
Proposed synergism between inflammatory stimulation and neurotrophic factors during multi-session treatment with electroconvulsive therapy (ECT). Before the first ECT session, depressed patients show reduced neurotrophin levels. An allostatic inflammatory response is thus endogenously triggered, mainly in the periphery, providing suboptimal inflammatory stimulation to the brain. Each ECT session strongly activates the innate immune system in the short term (minutes to hours post session) and thereby further mobilizes neurotrophin expression. Multiple inflammatory bouts are, however, needed over time (inter-session intervals of days to weeks) to achieve optimal neurotrophin availability. Upon remission, endogenous inflammation has no allostatic purpose and therefore resolves.

As described in this review, there is compelling evidence that ECT is closely related to an enhanced innate neuroinflammatory as well as haematogenous immune response. A related set of experimental evidence further shows that immune stimulation reinforces neurotrophin expression and possibly vice versa, thereby suggesting one possible route by which bouts of inflammation may mobilize endogenous neuroprotection (see Figure 1). However, we are far from understanding how such an effect could be ‘isolated' from the detrimental consequences of inflammation.

Overall, ECT and other electrically based CNS treatments may not only serve as drastic therapeutic modalities in psychiatry but may also represent an opportunity to study and possibly exploit the salutary facets of inflammation. To this end, both clinical and translationally relevant animal studies will be needed.
